# Discovery of Novel Liver-Stage Antimalarials through Quantum Similarity

**DOI:** 10.1371/journal.pone.0125593

**Published:** 2015-05-07

**Authors:** David J. Sullivan, Yi Liu, Bryan T. Mott, Nikola Kaludov, Martin N. Martinov

**Affiliations:** 1 W. Harry Feinstone Department of Molecular Micorbiology and Immunology, Johns Hopkins Bloomberg School of Public Health, Baltimore, Maryland, United States of America; 2 Division of Preclinical Innovation, National Center for Advancing Translational Sciences, National Institutes of Health, Rockville, MD, United States of America; 3 Gradient Biomodeling LLC, Park City, Utah, United States of America; Food and Drug Administration, UNITED STATES

## Abstract

Without quantum theory any understanding of molecular interactions is incomplete. In principal, chemistry, and even biology, can be fully derived from non-relativistic quantum mechanics. In practice, conventional quantum chemical calculations are computationally too intensive and time consuming to be useful for drug discovery on more than a limited basis. A previously described, original, quantum-based computational process for drug discovery and design bridges this gap between theory and practice, and allows the application of quantum methods to large-scale *in silico* identification of active compounds. Here, we show the results of this quantum-similarity approach applied to the discovery of novel liver-stage antimalarials. Testing of only five of the model-predicted compounds *in vitro* and *in vivo* hepatic stage drug inhibition assays with *P*. *berghei* identified four novel chemical structures representing three separate quantum classes of liver-stage antimalarials. All four compounds inhibited liver-stage *Plasmodium* as a single oral dose in the quantitative PCR mouse liver-stage sporozoites-challenge model. One of the newly identified compounds, cethromycin [ABT-773], a macrolide-quinoline hybrid, is a drug with an extensive (over 5,000 people) safety profile warranting its exploitation as a new weapon for the current effort of malaria eradication. The results of our molecular modeling exceed current state-of-the-art computational methods. Drug discovery through quantum similarity is data-driven, agnostic to any particular target or disease process that can evaluate multiple phenotypic, target-specific, or co-crystal structural data. This allows the incorporation of additional pharmacological requirements, as well as rapid exploration of novel chemical spaces for therapeutic applications.

## Introduction

Chemical similarity is a central principle in ligand design, and extensive chemoinformatic studies explore multiple methods based on it [1,[2]. However, chemical structure alone does not provide adequate description of bio-molecular interactions, which are quantum in nature. Through molecular modeling, molecules can be considered as quantum objects: quantum representation of their activity (biological, chemical or pharmacological), not the underlying structure itself, is important. Our quantum molecular representations exhibit well-defined mathematical characteristics, which afford systematic theoretical treatment and property prediction with methods that would otherwise be computationally impossible [3,[4]. Specialized machine-learning algorithms with fuzzy decision-making protocols [5] are then applied for retrospective data analysis to identify both active compounds and the corresponding quantum features of chemical and biological interest. The modeling data consists either of training sets of structurally diverse compounds with measured activity (EC_50_, IC_50_, K_d_ etc.) against the target or phenotype of interest, or of co-crystal structural data of the target and a modulator. Since structurally different entities can exhibit related quantum properties, the quantum representation of biological activity allows the identification of chemically dissimilar compounds, which are similar on a quantum level and vice versa. This feature facilitates the discovery of structurally novel active compounds, and has already been applied to blood stage antimalarial activity [3]. Here, new liver-stage quantum models were created based on experimental phenotypic data on compounds in a liver-stage *Plasmodium* bioassay for identifying prospective drugs [6].

## Materials and Methods

### Quantum comparison

#### Structure representation – localized electron-density descriptors for molecular modeling

Well-defined chemical subsystems, together with their associated local, spatially-resolved properties, are very useful in drug discovery [7]. On a theoretical level, these properties serve as powerful descriptors for molecular modeling and design. Notions from Density Functional Theory and Topological Theory of Atoms in Molecules can be combined to rigorously define and compute a complete set of such localized, electron-density descriptors.

In general, Non-Relativistic Quantum Mechanics provides the proper level of physical theory for treatment of molecular and bio-molecular systems [8]. However, many intuitive chemical concepts are not directly related to the corresponding wave function [9], a state-vector in Hilbert space, which is difficult to partition into chemically meaningful subsystems [10].

Density Functional Theory [11] provides a systematic framework for inferring chemistry-related information from quantum calculations. This is achieved through the use of the electron density, ρ(**r**), a real, nonnegative Cartesian function connected to the *N*-electron molecular wave function ψ by

$$\rho (r)=\int |\Psi (x,x_{1},\ldots ,x_{N-1}|{}^{2}\;dsdx_{1}\ldots dx_{N-1}$$

where **x** = {*s*, **r**} is the four-dimensional spin-spatial coordinate. As the famous Hohenberg-Kohn theorem [12] shows, ρ(**r**) determines all ground-state properties of the entire system, including its chemical and biochemical features.

Furthermore, the Topological Theory of Atoms in Molecules [13] uses ρ(**r**) to partition molecules into precise atomic subsystems. These atomic subsystems are bounded by zero-flux surfaces *S*, which obey the equation

∀r∈Sn(r)•∇ρ(r)=0

where **n**(**r**) is the vector normal to *S* at **r** and ρ(**r**) is the corresponding electron density.

It is natural to combine these two theories, together with their respective computational algorithms, in a single formalism for studying local molecular properties from first principles. This formalism has yielded meaningful interpretations of many general chemical concepts, such as energy partitioning [14], atomic softness [15], electronegativity equalization [16], atomic reactivity indices [17], etc. Augmented with the electrostatic potential, this electron density-based methodology has been applied to quantitative structure-activity relationship studies [18]. It also produced the molecular descriptors employed in the modeling effort described here. Importantly, when applied as descriptors, these electron-density transforms define a proper metric (molecular similarity measure) in the modeling space, and allow the use of rigorous mathematical techniques.

#### Modelling architecture—fuzzy decision networks

Molecular modeling is a multi-step process:

$$\left\{S_{i},P_{i}\right\}\to \left\{D_{i,j}\;\left(S\right),P_{i}\right\}\to P\left[D\left(S\right)\right]$$

The starting point, {*S*_*i*_, *P*_*i*_}, called a training set, is a set of molecular structures *S*_*i*_ for which a particular property of interest *P* has been measured. In the first step, descriptor calculation, every structure is reduced to some form, typically a list of real numbers {*D*_*j*_}, which can be modeled statistically. The second step, actual modeling, attempts to find a model—a general mapping between property *P* and structure *S* through descriptors *D*. If successful, the model would have predictive power that can be applied to structures for which no measurement exists. Naturally, the predictive power of the model depends on the quality (accuracy, diversity, etc.) of the training set as well as descriptor properties and modeling architecture.

Both powerful descriptors and proper modeling architecture are crucial for successful molecular modeling and compound discovery. Ideally, the modeling architecture should be chosen in accordance with the underlying fundamental processes of the system [19], and not with the type of available numerical data. Complex biochemical interactions involve local attributes of distinct and diverse molecular structures, which are best modeled with discrete combinatorial methods rather than continuous multivariate techniques. Still, inherent weaknesses of traditional molecular descriptors require the use of such continuous multivariate techniques [20]. As sophisticated as some of these techniques are, they cannot always compensate for the shortcomings of the underlying molecular-structure representations.

A straightforward machine-learning algorithm using fuzzy-logic decisions easily discovers the relationship between quantum components and specific interaction patterns. In its simplest implementation, the modeling algorithm produces a model in the form of a fuzzy decision tree [5]. Each tree node corresponds to a single descriptor (interaction constraint). In a fully resolved decision tree, terminal nodes contain only either active or inactive molecules. Furthermore, each terminal node is fully characterized statistically—if a molecule belongs to it, the prediction is qualified by associated confidence intervals and other statistical parameters. A model in the form of a decision tree is easy to interpret. Each tree path that contains an active terminal node also contains a set of nodes (quantum components) that define the interaction pattern common to all training-set molecules belonging to this terminal. The fuzzy decision tree formalism can be generalized to more powerful fuzzy decision algorithms. Given a diverse training set of structures with known inhibition, the modeling effort produces a decision network characterizing all present interaction patterns in terms of activity-controlling descriptors, which can be visualized.

### Synthesis of cethromycin and quality testing

Cethromycin in low milligram amounts was prepared according to literature procedure starting from commercially available erythromycin (Chem-Impex #00126) and quinoline (Sigma Aldrich-# 241571) [21]. The final compound was purified directly on silica gel using a Biotage Isolera One automated purification unit (0–16% methanol in dicholoromethane over 20 column volumes). The isolated compound was concentrated under reduced pressure and placed under high vacuum for two days: 15.0 mg, 63% yield (final step); ^1^H NMR (400 MHz, CDCl_3_) δ 9.03 (s, 1H), 8.18 (s, 1H), 8.06 (d, *J* = 8.4 Hz, 1H), 7.83 (d, *J* = 8.1 Hz, 1H), 7.64 (t, *J* = 7.6 Hz, 1H), 7.51 (t, *J* = 7.5 Hz, 1H), 6.56 (d, *J* = 16.0 Hz, 1H), 6.18 (dt, *J* = 15.7, 6.8 Hz, 1H), 5.55 (s, 1H), 4.93 (dd, *J* = 9.3, 3.1 Hz, 1H), 4.40 (d, *J* = 4.2 Hz, 1H), 4.38 (d, *J* = 6.4 Hz, 1H), 3.96 (q, *J* = 6.7 Hz, 1H), 3.90 (s, 1H), 3.84 (dd, *J* = 11.8, 6.6 Hz, 1H), 3.70 (dd, *J* = 11.9, 7.1 Hz, 1H), 3.62–3.51 (m, 1H), 3.33–3.22 (m, 1H), 3.23–3.14 (m, 1H), 2.96 (q, *J* = 6.5 Hz, 1H), 2.76–2.67 (m, 1H), 2.67–2.58 (m, 1H), 2.40 (s, 6H), 2.07 (bs, 1H), 1.92–1.85 (m, 1H), 1.83 (dd, *J* = 12.7, 5.5 Hz, 1H), 1.73 (d, *J* = 12.4 Hz, 2H), 1.67 (d, *J* = 13.8 Hz, 1H), 1.57–1.51 (m, 1H), 1.49 (s, 3H), 1.42 (s, 3H), 1.40 (d, *J* = 8.0 Hz, 3H), 1.38 (d, *J* = 6.6 Hz, 3H), 1.33–1.22 (m, 1H), 1.19 (d, *J* = 6.0 Hz, 3H), 1.14 (d, *J* = 7.8 Hz, 3H), 1.11 (d, *J* = 6.3 Hz, 3H), 0.79 (t, *J* = 7.4 Hz, 3H); ^13^C NMR (101 MHz, CDCl_3_) δ 217.39, 205.40, 169.67, 157.77, 149.59, 147.46, 132.66, 129.88, 129.17, 129.01, 128.58, 128.07, 126.81, 102.52, 83.55, 78.71, 77.52, 76.42, 69.99, 68.95, 66.05, 64.30, 58.22, 50.85, 46.22, 45.07, 39.99, 39.04, 37.34, 29.05, 27.90, 22.62, 21.09, 20.19, 18.08, 14.47, 14.11, 13.67, 10.66; LCMS—retention time = 3.747 min (4% to 100% Acetonitrile (0.05% TFA) over 7 minutes; Luna C18 column, 3 micron 3 x 75mm), MS (ESI) *m/z* (M+H)^+^ = 765.7; HRMS (ESI) for C_42_H_61_N_3_O_10_ *m/z* [(M+2H)/2]^+^ calculated = 383.7186, found = 383.7183. 15 mg of cethromycin was available for study.

### *In vitro P*. *berghei* liver stage assay

Drugs were purchased from Sigma (Tris(2-methylphenyl)tin—S819433 or Ambinter (T0507-9950- Amb356416, T5531873- Amb461470, T0510-7064- Amb1639503). In each chamber of 8-well LabTek tissue culture slides, 50,000 mouse hepatoma cells, Hepa1-6, were seeded one day before infection. Cells were normally cultured in DMEM supplemented with 10% FBS, 1X L-glutamine and 1X Pen-Strep at 37°C and 5% CO_2_. Approximately 60,000 *P*. *berghei* sporozoites from infected *Anopheles stephensi* obtained from the JHMRI parasite core laboratory, were added with media containing 2.5% FBS, 1X L-glutamine and 2X Pen-Strep. Before treatment with drugs 3 hours after addition of sporozoites, the cells were washed four times with DMEM containing 10X Pen-Strep and 5 ug/mL fungicide- Fungizone (Invitrogen/Gibco #15290–018) to prevent bacterial or fungal infection from the nonsterile sporozoite preparations, then returned to media with 2.5% FBS, 1X L-glutamine, 2X Pen-Strep and drugs. After 24 hours, fresh culture medium also containing drugs was exchanged in each chamber. Approximately 42 hours post-infection, the growth medium was removed and 100% cold methanol was added for 15 minutes to fix the cells. After washing with sterile PBS, cells were incubated in PBS with 5% FBS for blocking. Then 2E6 anti-HSP70 antibody was diluted to 10 μg/mL in PBS with 5% FBS and added in a 200 μL volume to cells. One hour later, cells were washed with PBS and incubated with 10 μg/mL Alexafour 594 anti-mouse secondary antibody. In order to visualize hepatocytes, nuclear counterstain DAPI was incubated with cells for 5 minutes and then washed off. The plastic chambers and silicone gasket were removed and slides were fully dried before mounted and sealed with cover slides. When preparation was complete, the slides were taken to the fluorescence microscope for examination with the 20x objective. Representative areas were randomly selected from each chamber and the numbers of infected cells were counted for a similar number of at least 3 microscopic fields representing more than 500 uninfected hepatocytes. The proportion of at least 500 Hepa1-6 cells that was infected in each well was measured, and the mean proportion of duplicate wells was calculated. Data from drug-treated samples were compared to those from control samples, which were normalized to 0%. The experiments were performed twice with two different mosquito sporozoite preparations.

### *In vivo P*. *berghei* liver stage assay

Mice were kept in Johns Hopkins Bloomberg School of Public Health mouse facility according to the approved Johns Hopkins University ACUC animal protocol number MO09H401. The animal work was carried out in strict accordance with the recommendations in the Guide for the Care and Use of Laboratory Animals of the National Institutes of Health. All efforts were made to minimize suffering. Six week old C57BL/6 mice weighing about 20–22 g were divided into groups of three. 12,000 *P*. *berghei* sporozoites in a 200 μL volume were injected into mice through tail veins. Two hours after injection, different dilutions of drugs in 100–200 μL volumes were delivered to mice by oral gavage using a plastic feeding tube. A second dose of drugs was given 24 hours after the first one to the mice that were on a daily dosing regimen. Approximately 40 hours post-infection, mice infected with *P*. *berghei* sporozoites were anesthetized ketamine injection, followed by cervical dislocation and sacrificed for harvesting whole livers. Each mouse liver was immediately put into 10 mL of Trizol Reagent and fully homogenized. After RNA isolation, the RNA was diluted to 100 ng/mL for reverse-transcription reactions. For a 30 μL reaction, the components were set up as: 3.5 μL of nuclease-free water, 3 μL of 10X Buffer II and 10 mM dNTPs, 6 μL of MgCl_2_ solution, 1.5 μL of 50 μM Random Hexamers, RNase Inhibitor and MuLV Reverse Transcriptase, 10 μL of RNA sample. cDNA products were stored at -20°C. For a 10 μL real-time quantitative PCR, the components were set up as: 0.2 μL of 10 μM forward and reverse primer 18s *P*. *berghei*, 5 μL of 2X SYBR Green PCR Master Mix, 1.6 μL of nuclease-free water, and 3 μL of cDNA sample. All test subjects were done in duplicates including positive and negative controls. When real-time PCR was finished, all data were baselined and normalized to the housekeeping gene HPRT before further analysis. The specific sequences of primers (The Core DNA Analysis Facility—JHU, Baltimore, MD) were: 5’- GGAGATTGGTTTTGACGTTTATGCG-3’ and 5’- AAGCATTAAATAAAGCGAATACATCCTTA-3’ for *P*. *berghei* ANKA 18s and 5’- TCCCAGCGTCGTGATTAGC-3’ and 5’- CGGCATAATGATTAGGTATACAAAACA-3’ for mouse HPRT.

## Results

**Fig 1:**
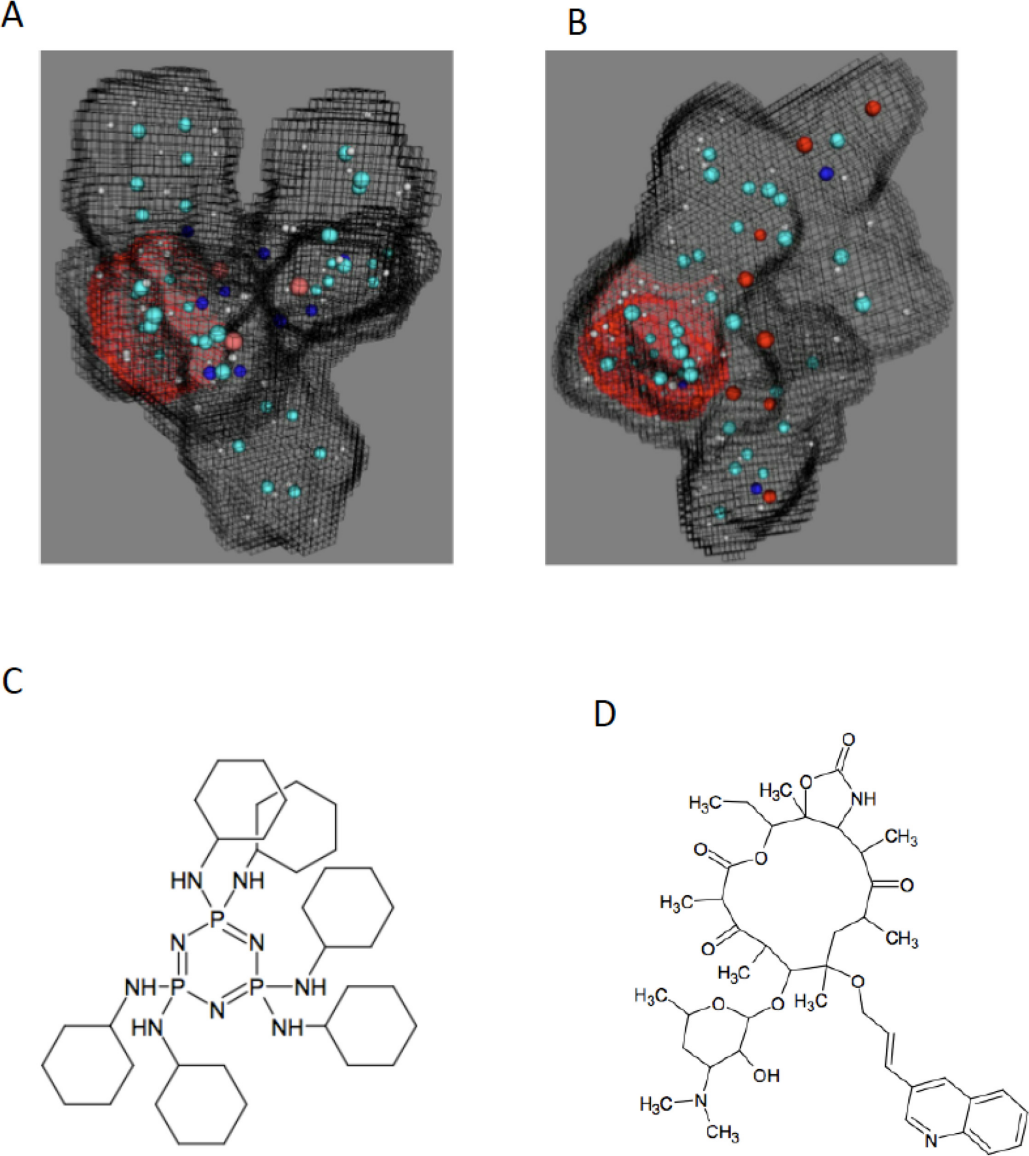
Liver-stage Quantum Components. Quantum similarity of **A.** GNF-Pf-1498 and the quinoline-macrolide hybrid **B.** cethromycin that is related to CHEMBL440116 as well as chemical structures of **C.** GNF-Pf-1498 and **D.** cethromycin.

**Fig 2:**
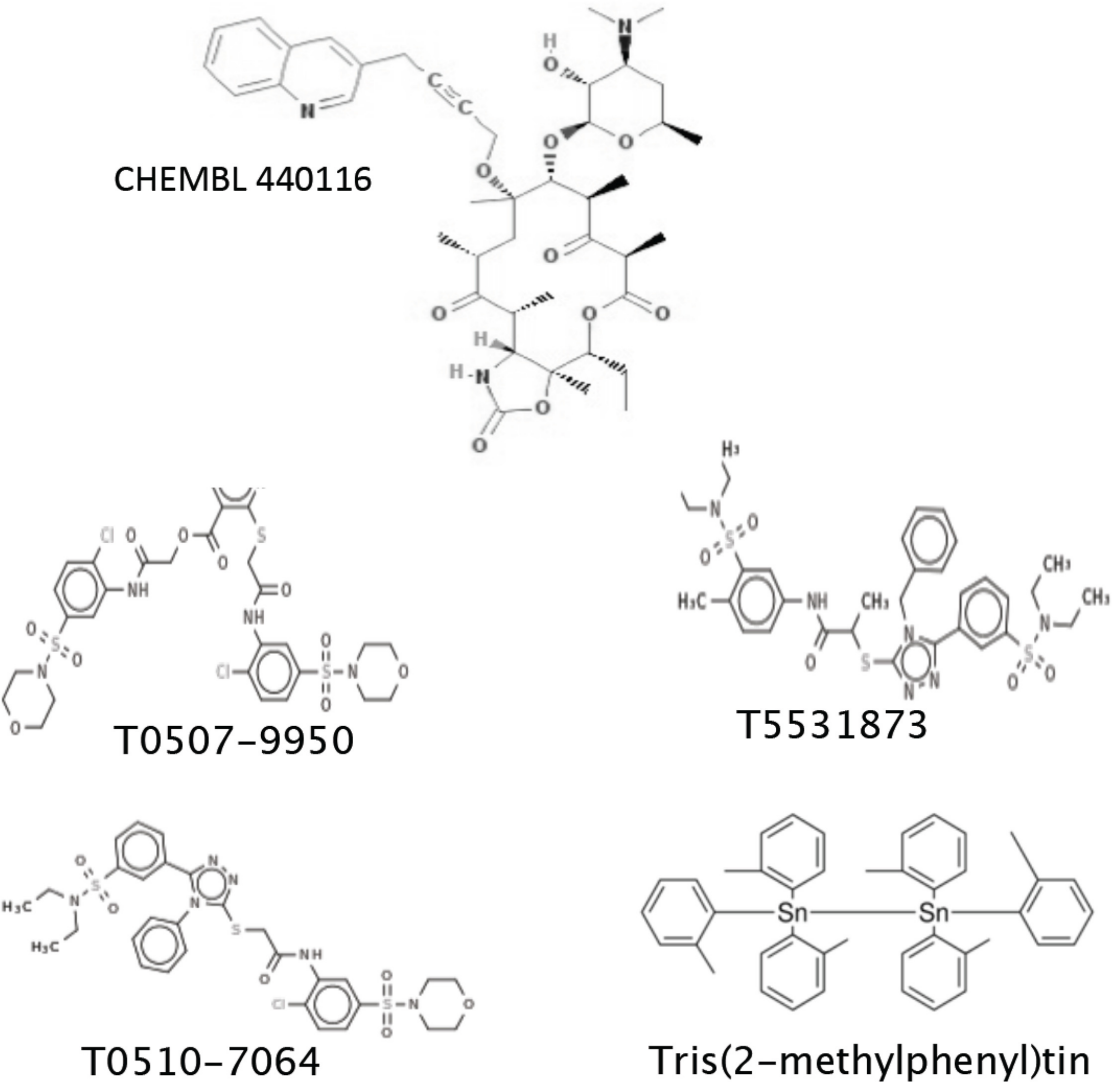
Compound Structures from Quantum modeling. Chemical structures of CHEMBL440116 and four other identified molecules which were acquired and tested.

**Fig 3:**
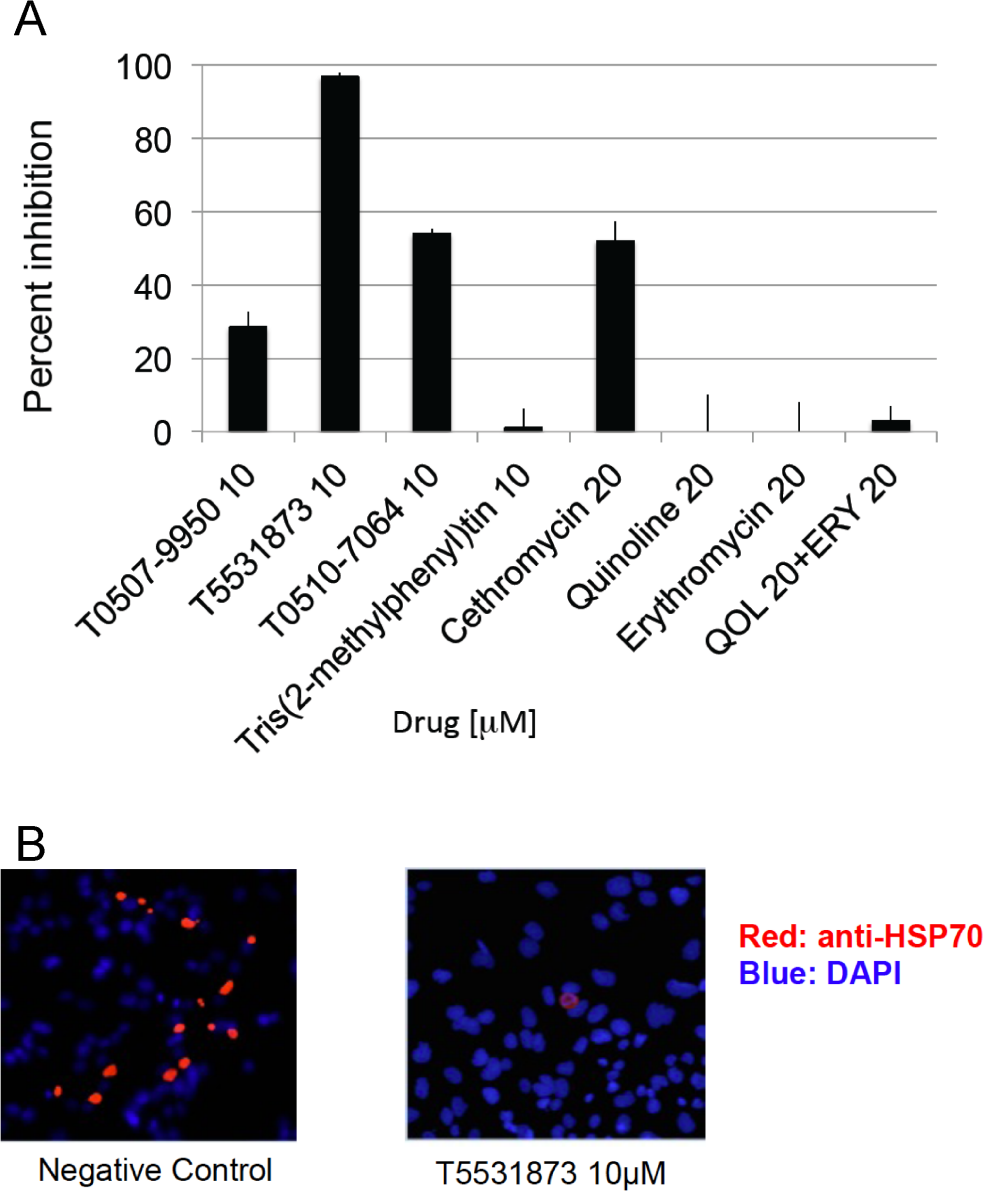
*in vitro* inhibition of liver stage malaria. **A.** Three of the new compounds indicated 30%, 96% and 55% inhibition while cethromycin alone at 20 μM had 54% inhibition. The individual components of cethromycin-quinoline and erythromycin, were inactive. Error is average of duplicate wells performed in two independent biologic experiments with different preparations of sporozoites. **B**. Microscopic image taken at 20x magnification of near 95% inhibition by T5531873. 50,000 Hepa1-6 cells were seeded in each well 24 hrs prior to infection with ~60,000 *P*. *berghei* sporozoites. The 2E6 anti-HSP70 antibody was used for immunofluorescent numeration of infected cells.

A training dataset of 5757 compounds was generated by combining *P*. *yoelii* liver stage data from Novartis ChEMBL-NTD HTS [6] and additional validated liver stage antimalarial drugs from recent publications that investigated *in vitro* hepatocyte *P*. *berghei* inhibition [6,[22,[23,[24]. The dataset was utilized to establish the quantum components (QCs) related to liver-stage inhibition. These liver-stage QCs were used as filters to virtually screen a database of over 65 million commercially available compounds, which were already pre-computed in a quantum format suitable for fast processing. This *in silico* screen was followed by a chemical-diversity filter to assure that the identified compounds are novel and chemically different from the training set. Since QCs related to a specific biological or other activity can be carried by structurally different compounds [3,[4], the procedure calculated the quantum similarity between the QCs established from the liver-stage training set modeling and the QCs of the commercially available compounds in the database. This process identified and rank-ordered 35 different, chemically dissimilar from the training set structures, predicted by the models to be active based on their similar liver-stage activity QCs. A number of commonly accepted theoretical measures of chemical similarity [25] were considered to estimate the novelty of the proposed compounds. These include Tanimoto coefficients [26] based on pharmacological functional groups or compound fragments [27], as well as chemical diversity measures derived from electron density considerations [5]. Once computed, these indices were used to create point-to-set distance metrics [28], which determine the dissimilarity of the considered structure from the known liver-stage active molecules (Fig 1). The model matches the input molecules with identified candidates over numerous quantum scoring criteria. A representative image of matching the quantum components of model-predicted cethromycin and training set molecule GNF-Pf-1498 [6] is shown in Fig 1. The red region demonstrates rigorous theoretical quantum similarity between the non-macrolide quinoline on cethromycin and the nitrogen-rich aromatic ring of GNF-Pf-1498 with respect to liver stage activity. Tables 1 and 2 identifies the matched quantum score with the reference input molecules from the training set. Reference molecules Cyclosporin A and monensin have a liver stage inhibition of 1.7 nM [23] and 0.001 nM [24] respectively. Cethromycin is chemically similar to one of the top potential candidates, bearing an allylic linker between the macrolide and the quinoline (versus a four-carbon linker containing a triple bond seen in CHEMBL 440116 in Fig 2). The structures of the other four obtained compounds are shown in Fig 2, and quantum scores and identification are in Table 3. Cethromycin was directly synthesized from erythromycin by substituting the cladinose sugar at C3 with a keto-group, attaching a cyclic carbamate group at C11-C12, and tethering the quinoline moiety to the C6 alcohol [21]. We used the *in vitro P*. *berghei* ANKA model on mouse hepatoma cells, Hepa1-6. Among all drugs and compounds tested, T5531873 at 10 μM was able to reduce parasite multiplication by over 95%, while cethromycin at 20 μM and T0510-7064 at 10 μM reduced growth by 52% and 54%, respectively (Fig 3). Quinoline, erythromycin and their combination were also tested to investigate the role the quinoline plays in the antimalarial activity of cethromycin. Neither quinoline, nor erythromycin, nor their combination showed any parasite inhibition *in vitro* (Fig 3) Of the five compounds tested *in vitro*, those that demonstrated measurable inhibitory effect on parasite viability in the hepatoma cells (T0507-9950, T5531873, T0510-7064 and cethromycin) were further validated in a mouse model at a dose of 50 mg/kg. Interestingly, the five compounds with predicted *in vitro* activity belong to four distinct quantum classes (Table 3). Since four of these predicted compounds are active *in vitro* (80% success rate), the three quantum classes corresponding to them are experimentally validated and can be used for future design of new chemical entities with optimized anti-malarial properties. The remaining quantum class, which contains Tris(2-methylphenyl)tin and Everolimus, is not validated by this experiment. This is not surprising considering that the modeling only provides some of the necessary quantum constraints controlling molecular properties. The modeling information content is limited by the quantum data present in the training set, and cannot be expected to capture all possible reasons for non-activity. For example, one explanation is that Tris(2-methylphenyl)tin is not accessible to the cytosolic compartment required to inhibit the *Plasmodium*, and this information was not adequately represented by the modeling data.

**Table 1:** Matching quantum scores of input and output molecules from cyclosporine.

Input	Cyclosporin A	0	0	0	1(0;1)(1)	1(1;1)(1)	0	1(1;1)(3)	1(1;1)(4)	0	1(1;2)(1;2)
Output	T5531873	1(1;1)(1)	1(1;1)(1)	1(1;1)(1)	1(0;1)(1)	1(0;1)(1)	0	1(1;1)(3)	1(0;5)(1;2;3;4;5)	0	1(0;2)(1;2)
Output	T0510-7064	1(1;1)(1)	1(1;1)(1)	1(1;1)(1)	1(0;1)(1)		0	1(1;1)(3)	1(0;5)(1;2;3;4;5)	0	1(0;2)(1;2)

**Table 2:** Matching quantum scores of input and output molecules from monesin and telithromycin.

**Input**	**Monensin**	**0**	**0**	**0**	**1(0;1)(1)**	**1(1;1)(1)**	**0**	**1(1;1)(1)**	**1(1;1)(1)**	**0**	**1(1;2)(1;2)**
**Input**	**Telithromycin**	**0**	**0**	**0**	**1(0;1)(1)**	**1(1;1)(1)**	**1(1;1)(1)**	**1(1;1)(1)**	**1(1;1)(1)**	**0**	**1(1;2)(1;2)**
Output	CHEMBL440116	0	0	0	1(0;1)(1)	1(1;1)(1)	1(1;1)(1)	1(1;1)(1)	1(1;1)(1)	0	1(1;2)(1;2)

**Table 3:** Summary of quantum scores with molecule identifications and reference molecules for scoring.

Name	Quantum pattern score	Reference molecule	CID
T0507-9950	19	Cyclosporin A	5109719
T5531873	21	Cyclosporin A	16290646
T0510-7064	20	None	5011386
Tris(2-methylphenyl)tin	18	Everolimus	50932585
CHEMBL440116	15	GNF-Pf-1498; Monensin	44296067
Cethromycin		GNF-Pf-1498; Monensin	447451

**Fig 4:**
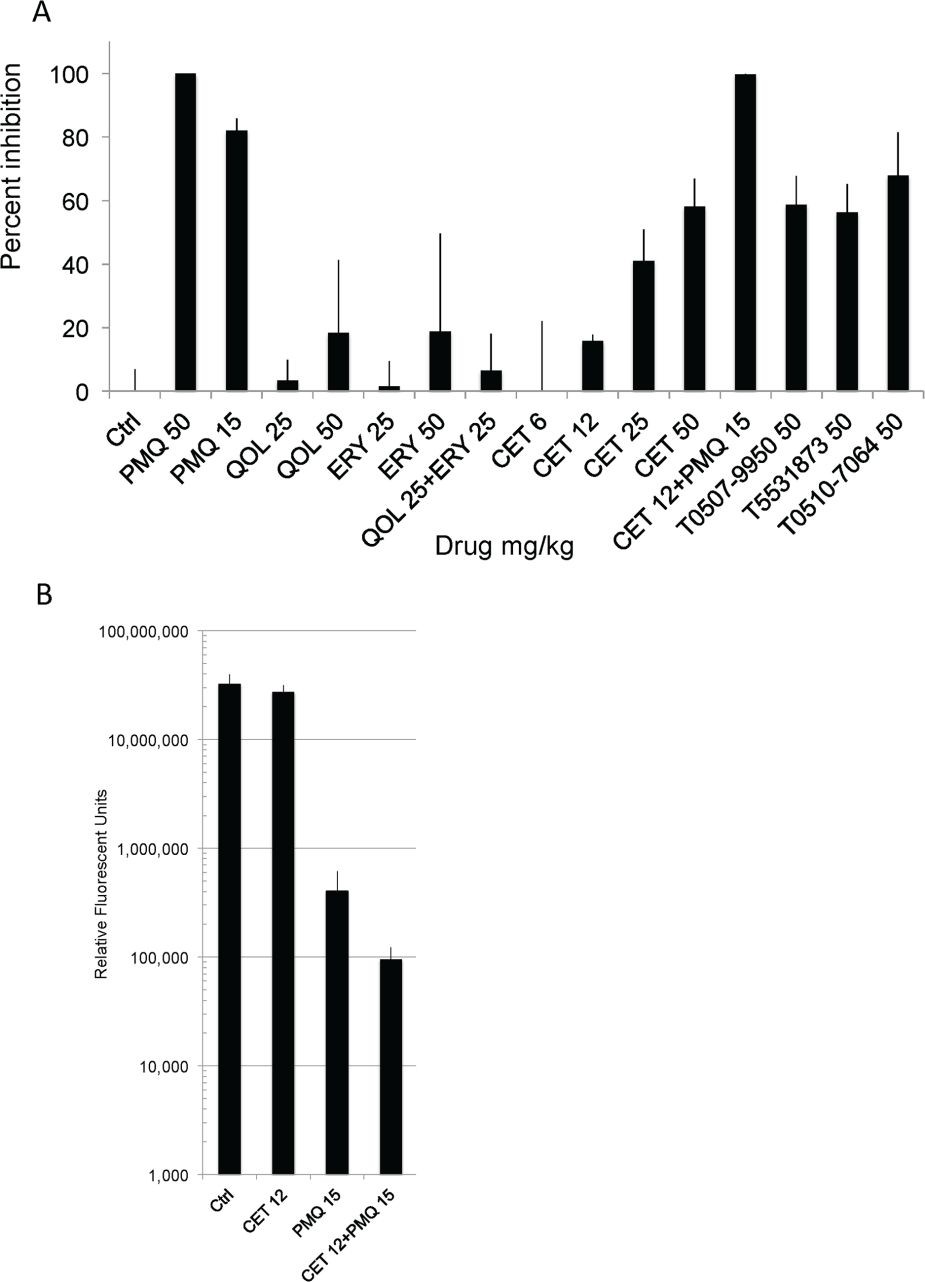
*in vivo* inhibition. 10,000 sporozoites were inoculated by tailvein injection and mice were sacrificed 40 hours later, livers were harvested, placed in RNAzol and parasite levels determined by realtime PCR from cDNA from reverse transcription. Relative fluorescent units were compared to control to determine percent inhibition. Cethromycin CET was administered only once while the other drugs were given twice 24 hours apart from each dose. Two drugs related to CET, quinolone (QN) and erythromycin (ERY), had only marginal effect on parasite growth. CET’s effectiveness increased with dosage, reaching 60% reduction at 50 mg/kg. CET was also able to eliminate parasite infection when combined with low dose of PQ. All three novel compounds (T0507-9950, T5531873, T0510-7064) demonstrated significant inhibitory effect on parasite proliferation. Error is standard error of mean of three mice with real time PCR performed in duplicate for transcript levels in each mouse. B. Actual log values of relative fluorescent units from controls, cethromycin 12 mg/kg, primaquine 15 mg/kg and in combination are depicted. The combination has a 4 fold drop in relative fluorescent units compared to primaquine.

Consistent with results from the *in vitro* assay, quinoline, erythromycin and their combination measured only marginal inhibitory effect on parasite replication shown in Fig 4A. Although cethromycin at 12 mg/kg was only partially effective, a gradual dose response was observed after increasing the dose to 50 mg/kg, at which cethromycin reduced parasite load by 60 percent. Due to limited synthesized amounts of cethromycin, only one dose was given to mice while other drugs and compounds were administered twice. We were able to achieve near parasite elimination by combining cethromycin at a dose of 12 mg/kg with primaquine at a dose of 15 mg/kg. While cethromycin at 12 mg/kg showed minimal inhibition at about 16%, combination with low dose primaquine at 15 mg/kg (itself achieving 98% inhibition) showed greater than 99% inhibition. The log reduction these two drugs and in combination is shown in Fig 4B. Although the T5531873 compound was not as effective as in the *in vitro* assay, all three commercial compounds (T0507-9950, T5531873 and T0510-7064) reduced parasite load by more than 50 percent without causing any notable side effects in the mice after two doses. Based on these results and established human clinical data, cethromycin has the most potential of the tested compounds for early clinical malaria liver studies.

## Discussion

The past decade has witnessed unprecedented efforts in global control of malaria. However, when aiming for elimination and eradication, the whole spectrum of human pathogenic *Plasmodium* need to be considered, with a particular emphasis on *P*. *vivax*, whose public health impact has been largely underestimated. Currently, curative treatment for *P*. *vivax* that is dormant in the liver stage relies on a 14-day course of an 8-aminoquinoline, primaquine, which is contraindicated for individuals with severe glucose-6-phosphate dehydrogenase deficiency. Research and development efforts into new chemical entities to treat dormant *P*. *vivax* effectively with a single dose or a few oral doses are urgently needed if elimination efforts are to become a reality.

Our scientific objective was to identify novel liver-stage antimalarials, which could later be tried for dormant *P*. *vivax*, using a previously described and experimentally validated drug discovery methodology [3,4] that is data-driven and broad-spectrum in biological focus, unbiased in its consideration of prior disease-specific molecular interactions and computationally-enabled by formal models of disease pathways and host-pathogen mechanisms. This methodology delivers testable, evidence-based hypotheses suitable for rapid experimental exploration of novel chemical space for therapeutic applications. It identifies and prioritizes candidate therapeutic compounds based on the similarity between the QCs established from the liver-stage training set modeling and the QCs of the commercially available compounds in our pre-computed database. Using the quantum similarity modeling approach, we discovered a drug candidate, ready to be evaluated in humans for malaria elimination and identified several additional orally bioavailable lead molecules that inhibited the parasite in an animal liver-stage model. Empirical validation of predicted compounds in cellular and mouse model systems documented an effective prediction rate of over 66%, suggesting the utility of such computational discovery approaches.

One of the newly identified compounds, Cethromycin [ABT 773], a macrolide-quinoline hybrid, is a drug with extensive safety profile that was active with more than a log decrease in mice which in addition was at least additive with primaquine. Cethromycin is an erythromycin and quinoline nucleus hybrid. Individually [29] erythromycin and quinoline have no activity *in vitro* or in the mouse model, but cethromycin was active. Cethromycin has been used safely in over 5,000 humans in efficacy studies for single day dosing for bacterial pneumonia [30]. Progression to the market was halted after the FDA requested more efficacy data in 2009 [31]. A recent clinical pneumonia trial showed that cethromycin was non-inferior to clarithromycin [32]. The safety and pharmacokinetics are a suitable match for a potential safe effective human liver stage malaria drug. With a single dose of 300 mg each day the volume of distribution for cethromycin was 769 mL/kg with plasma Cmax of 0.5 mcg/mL (~600 nM) and half-life of 5 hours with an AUC of 3 mcg/h/mL [29,[33]. Alveolar levels were measured at 55 mcg/mL (~60 μM) with a longer half-life of 11 hours. [29,[33] The drug is eliminated in the liver and is predicted to have higher liver levels than plasma although this has not been formally measured. The single daily oral dose of 300 mg cethromycin in humans translates to 60 mg/kg in humans with the 600 mg dose equal to 120 mg/kg in the mouse. This is based on the meter square dosing originating with cancer chemotherapy [34]. Because of limited drug we were not able to push to normal human equivalent dosing. This suggests that the favorable pharmacokinetics of cethromycin predict liver stage pharmacodynamics in the μM range in human liver *Plasmodium* stages suitable for either malaria prevention or possible radical cure of dormant *P*. *vivax* or *P*. *ovale* hypnozoite stages.

The research described here was performed in less than a year, at a fraction of the cost of similar drug discovery efforts [6]. The existing commercial compounds T0507-9950, T5531873 and T0510-7064 could serve as excellent leads in a traditional medicinal chemistry optimization. Equally importantly, however, the model-discovered liver-stage quantum properties have been experimentally validated. Furthermore, since this type of molecular modeling does not depend explicitly on chemical structures but on their quantum components that can be shared by multiple chemically different compounds, quantum similarity allows the design of novel chemical entities with simultaneously optimized target activity, therapeutic efficacy and other favorable pharmacological characteristics. Once a quantum model has been validated, it provides a powerful tool for *de novo* lead discovery by inverse molecular construction, which combines on the same chemical structure the identified quantum molecular attributes responsible for liver-stage antimalarial activity and other physicochemical properties of interest. Their synthesis and experimental evaluation is ongoing.

In conclusion, we suggest that our systems biology pathway models can be used alongside traditional screening-based approaches in drug discovery initiatives. Our work demonstrates the potential of theoretical models to enable rapid, systematic identification and prioritization of novel compounds against existing or emerging biological threats, thus accelerating therapeutic and medical countermeasures research.
